# Supplementary material for: Full Dilatation Caesarean Section and the Risk of Preterm Delivery in a Subsequent Pregnancy: A Historical Cohort Study
Source: J Clin Med. 2020 Dec 10;9(12):3998. doi: 10.3390/jcm9123998 (PMC7763432; doi:10.3390/jcm9123998)
Supplement: Supplementary file 1 [file jcm-09-03998-s001.pdf]

Original Research Manuscript

Full Dilatation Caesarean Section and the Risk of Preterm Delivery in a Subsequent Pregnancy: A Historical Cohort Study

Lauren Jade Ewington[1,2*] and Siobhan Quenby[1,2]

Supplementary Material

Supplementary Table S1. Additional baseline characteristics of the study population.

		Cervical Dilatation at Index Caesarean Section			
		0-5cm (n=707) n (%)	6-9cm (n=423) n (%)	Full (n=329) n (%)	*P*
**History of second trimester miscarriage**	Yes	6 (0.8)	5 (1.2)	1 (0.3)	0.42
	No	701 (99.2)	418 (98.8)	328 (99.7)	
**History of domestic violence**	Yes	7 (1.0)	4 (0.9)	5 (1.5)	0.70
	No	700 (99.0)	419 (99.1)	324 (98.5)	
**History of drug use**	Yes	9 (1.3)	4 (0.9)	2 (0.6)	0.60
	No	697 (98.7)	419 (99.1)	327 (99.4)	
**History of large loop excision of transformation zone**	Yes	8 (1.1)	2 (0.5)	6 (1.8)	0.21
	No	699 (98.9)	421 (99.5)	323 (98.2)	
**History of essential hypertension**	Yes	11 (1.6)	5 (1.2)	3 (0.9)	0.67
	No	696 (98.4)	418 (98.8)	326 (99.1)	
**Pregnancy induced hypertension in the index pregnancy**	Yes	37 (5.2)	13 (3.1)	9 (2.7)	0.08
	No	670 (94.8)	410 (96.9)	320 (97.3)	
**Pre-eclampsia in the index pregnancy**	Yes	50 (7.1)	18 (4.3)	17 (5.2)	0.13
	No	657 (92.9)	405 (95.7)	312 (94.8)	
**Duration of active phase of index pregnancy labour (hours)**	<6	433 (61.2)	71 (16.8)	26 (7.9)	<0.001
	6-11	157 (22.2)	129 (30.5)	122 (37.1)	
	12-17	94 (13.3)	174 (41.1)	126 (38.3)	
	18-23	19 (2.7)	46 (10.9)	46 (14.0)	
	≥24	4 (0.6)	3 (0.7)	9 (2.7)	
**Estimated blood loss at index caesarean section (mls)**	<499	226 (33.4)	103 (26.1)	67 (21.5)	0.002
	500-999	351 (51.9)	209 (53.0)	188 (60.5)	
	1000-1499	72 (10.7)	60 (15.2)	41 (13.2)	
	≥1500	27 (4.0)	22 (1.6)	15 (4.8)	
**Birthweight index neonate (Kg)**	<2.5	46 (6.5)	5 (1.2)	3 (0.9)	<0.001
	2.5-3.9	569 (80.5)	331 (78.3)	260 (79.0)	
	≥4.0	92 (13.0)	87 (20.6)	66 (20.1)	

Supplementary Table S2. Odds ratio for all baseline variables thought to increase the risk of spontaneous preterm birth in a subsequent pregnancy. sPTB: Spontaneous preterm birth.

		sPTB <37 weeks' gestation (n)	Delivery ≥37weeks' gestation (n)	Odds ratio (95% CI)	*P*
**Age**	<18	1	20	1.20* (0.11 - 6.88)	0.58
	18 - 34	53	1271	1.00	
	≥ 35	5	109	1.1* (0.46 – 2.70)	0.80
**Ethnicity**	White	34	919	1.00	
	Black	7	191	0.99 (0.42 – 2.17)	0.98
	Asian	16	232	1.86 (1.01 - 3.35)	0.04
	Other	2	58	0.93* (0.22 – 3.38)	>0.99
**Body mass index**	<18.5	2	31	0.74* (0.39 – 1.48)	0.37
	18.5 - 24.9	26	665	1.00	
	25.0 - 29.9	12	413	0.73 (0.38 – 1.43)	0.40
	≥30.0	19	290	1.68 (0.90 – 3.10)	0.09
**Parity**	Multiparous	14	244	1.00	
	Primgravida	45	1166	0.67 (0.36 – 1.25)	0.21
**Previous miscarriage 12-24 weeks'**	No	59	1388	1.00	
	Yes	0	12	0.00* (0.00 - 7.90)	>0.99
**Previous caesarean section**	No	54	1309	1.00	
	Yes	5	91	1.33* (0.52 - 3.41)	0.35
**Smoker**	No	46	1198	1.00	
	Yes	13	202	1.68 (0.89 - 3.15)	0.11
**History of domestic violence**	No	59	1384	1.00	
	Yes	0	16	0.00* (0.00 - 5.49)	>0.99
**History of drug use**	No	57	1386	1.00	
	Yes	2	13	3.74 (0.82-16.98)	0.09
**History of large loop excision of transformation zone**	No	59	1384	1.00	
	Yes	0	16	0.00* (0.00 - 5.49)	>0.99
**History of essential hypertension**	No	59	1381	1.00	
	Yes	0	19	0.00* (0.0 - 4.47)	>0.99
**Pregnancy induced hypertension in the index pregnancy**	No	54	1346	1.00	
	Yes	5	54	2.3* (0.96 - 5.58)	0.09
**Pre-eclampsia in the index pregnancy**	No	57	1317	1.00	
	Yes	2	83	0.56* (0.13 - 2.05)	0.58
**Induction of labour in index pregnancy**	No	32	652	1.00	
	Yes	27	748	0.74 (0.44 - 1.26)	0.74
**Duration of active phase of index pregnancy labor (hours)**	>12	17	504	1.00	
	<12	42	896	1.39 (0.78 - 2.47)	0.26
**Indication for index caesarean section**	Fetal distress	25	551	1.00	
	Failure to progress	26	671	0.85 (0.49 - 1.50)	0.58
	Other	8	178	0.99 (0.44 - 2.24)	0.96

* Fishers Exact test
